# Supplementary material for: Static versus dynamic muscle modelling in extinct species: a biomechanical case study of the Australopithecus afarensis pelvis and lower extremity
Source: PeerJ. 2024 Jan 31;12:e16821. doi: 10.7717/peerj.16821 (PMC10838096; doi:10.7717/peerj.16821)
Supplement: Supplemental Information 1 [file peerj-12-16821-s001.docx]

**Supplementary Information 1.**

**Musculoskeletal model creation**

A modern human (henceforth, just ‘human’) musculoskeletal model was used here to evaluate the AL 288-1 models (n=1, specimen ID: Subject03) (Charles et al., 2020), which was previously modified to include additional muscle-units (Wiseman, 2023). Additional muscles were appended to the lower limb, discussed in detail by Wiseman (2023). Subject03 was a young, adult female aged 26, weighing 72.6 kg and 176 cm in height. The model includes 36 muscles of the right side of the pelvis and right lower limb, crossing the hip, knee, ankle and metatarsophalangeal joints (Table 1). The Wiseman (Wiseman, 2023) *Australopithecus afarensis* model was used, which is based upon the AL 288-1 specimen, with the foot belonging to specimen AL 333-115 (DeSilva et al., 2018). Musculature for this model was digitally reconstructed following a 3D polygonal modelling approach (Demuth et al., 2022) and has previously been described in detail (Wiseman, 2023). Briefly, this method was implemented in Autodesk Maya 2022 (Autodesk Inc., San Rafael, USA), creating volumetric reconstructions of muscle-tendon units, with a line of action threaded through the centroid of each muscle cross-section. Such reconstructions are guided by muscle scarring where visible, cross-sectional data (here, magnetic resonance imaging scans of a modern human which were aligned by diaphyseal shaft diameter), and also by comparative dissection data (see: Wiseman 2023). In total, this produced 36 3D muscle geometries of the AL 288-1 pelvis and lower limb (Table 1) – this total does not differentiate between muscles composed of multiple heads (i.e., the EDL).

A convex hull approach for estimating inertial properties (mass, centre of mass (COM) and inertial tensors) of the body segments in the AL 288-1 model (Brassey et al., 2018, Coatham et al., 2021) was conducted in MATLAB 2021a (MathWorks, Natick) as described and implemented by Wiseman (2023). A convex hull wraps around a body as close to the segments as possible, and thus may exclude the space occupied by fatty deposits and other soft tissues. Therefore, correction factors from Coatham et al. (2021) were applied to each of these estimates to amend potential underestimates in the convex hull approach – see further details in Wiseman (2023). The musculoskeletal model was generated in OpenSim 4.3 (Delp et al., 2007, Seth et al., 2018).

*Additional references:*

COATHAM, S. J., SELLERS, W. I. & PUSCHEL, T. A. 2021. Convex hull estimation of mammalian body segment parameters. *Royal Society Open Science,* 8**,** 210836.
