# Supplementary material for: Static versus dynamic muscle modelling in extinct species: a biomechanical case study of the Australopithecus afarensis pelvis and lower extremity
Source: PeerJ. 2024 Jan 31;12:e16821. doi: 10.7717/peerj.16821 (PMC10838096; doi:10.7717/peerj.16821)
Supplement: Supplemental Information 3 [file peerj-12-16821-s003.docx]

**Supplementary Information 2.**

Joint angles (degrees) used in each of the quasi-static inverse simulations. All values in italics were joint angles that were positioned in a mid-stance posture and then subsequently locked. Both the human and australopith used the same joint angles in the left limb.

Changes in joint angles between the human and AL 288-1 are marked by an asterisk (*).

|  | **Human** | | **AL 288-1** |
| --- | --- | --- | --- |
| **Joint Rotation** | **Right limb** | **Left limb** | **Right limb** |
| Hip Flexion-Extension | 6.617 | *27.070* | 6.617 |
| Hip Adduction-Abduction | 0.071* | *-9.256* | 3.000* |
| Hip Rotation | -3.111 | *1.421* | -3.111 |
| Knee Flexion-Extension | -7.087 | *-61.954* | -7.087 |
| Knee Adduction-Abduction | *3.881** | *-7.651* | *-6.585** |
| Knee Rotation | *-4.278* | *-10.166* | *-4.278* |
| Ankle Dorsiflexion-Plantarflexion | -5.510 | *7.347* | -5.510 |
| Ankle Adduction-Abduction | *4.597* | *-7.113* | *4.597* |
| Ankle Inversion-Eversion | *-1.636* | *12.150* | *-1.636* |
| MTP Flexion-Extension | 20.387 | *-27.952* | 20.387 |
