# Supplementary material for: Static versus dynamic muscle modelling in extinct species: a biomechanical case study of the Australopithecus afarensis pelvis and lower extremity
Source: PeerJ. 2024 Jan 31;12:e16821. doi: 10.7717/peerj.16821 (PMC10838096; doi:10.7717/peerj.16821)
Supplement: Supplemental Information 4 [file peerj-12-16821-s004.docx]

**Supplementary Information 3**.

‘Residual actuators’ at the pelvis-body segment in the human and AL 288-1 simulations. Relative values are in brackets. Residual forces were all <5% of the maximum GRF magnitude (values proportional to the applied GRF, but differences were minute; here the residual values are reported for the 1 * BW simulation) and the residual moments were <1% of the height of the COM * maximum GRF.

|  | $\boldsymbol{Fx}$ | $\boldsymbol{Fy}$ | $\boldsymbol{Fz}$ | $\boldsymbol{Mx}$ | $\boldsymbol{My}$ | $\boldsymbol{Mz}$ |
| --- | --- | --- | --- | --- | --- | --- |
| **Human** | 7.365  (1.0%) | 0.437  (0.062%) | 0.436  (0.061%) | -3.590 (0.50%) | 0.432 (0.061%) | -44.350 (0.62%) |
| **AL 288-1** | 3.798 (1.1%) | 0.523 (0.15%) | 0.224  (0.066%) | 3.052 (0.90%) | 0.331  (0.098%) | -2.934  (0.86%) |
