# Supplementary material for: Static versus dynamic muscle modelling in extinct species: a biomechanical case study of the Australopithecus afarensis pelvis and lower extremity
Source: PeerJ. 2024 Jan 31;12:e16821. doi: 10.7717/peerj.16821 (PMC10838096; doi:10.7717/peerj.16821)

**Supplementary Information 5**

Muscle activations in the human musculoskeletal model in this study are reported at 30% of the gait cycle in comparison to published electromyography (EMG) studies. Here, simulated muscle activations as per the static optimisation outputs are used. No muscle activation dynamics have been modelled and, in this approach, simulated excitations are not produced.

EMG results across the gait cycle were collected from published studies (Cappellini et al., 2006, Wall-Scheffler et al., 2010, Perry and Burnfield, 2010, Van Criekinge et al., 2018) and are reported in red in the below table (next page). Here, we separate muscle excitations between stance (0-60%) and swing (60-100%) phases of the gait cycle. The simulated activations from the inverse simulations of the human model are reported in black at 30% of the gait cycle (marked by the vertical black dashed line in the table). We do not report on activation magnitudes here.

There are three notable differences between our simulations and published studies. First, the SM muscle is activated during stance but is predominantly a swing-phase muscle. Second, the EDLs are active but should be inactive, and the FDLs are inactive but should be active. Regarding the EDLs, these were minimally activated in our study in which we selected a cut-off activation of 0.045 to denote active versus inactive muscles. Raising this value to 0.155 changed these muscles to be inactive. We thus consider the EDL activation as inconsequential. It is questionable whether the POP and PL should be expected to be excited or active at mid-stance; our simulations did not obtain such activation.

CAPPELLINI, G., IVANENKO, Y. P., POPPELE, R. E. & LACQUANITI, F. 2006. Motor patterns in human walking and running. *J Neurophysiol,* 95**,** 3426-37.

PERRY, J. & BURNFIELD, J. M. 2010. *Gait Analysis: Normal and Pathological Function,* New York, NY, Slack Inc.

VAN CRIEKINGE, T., SAEYS, W., HALLEMANS, A., VAN DE WALLE, P., VEREECK, L., DE HERTOGH, W. & TRUIJEN, S. 2018. Age-related differences in muscle activity patterns during walking in healthy individuals. *J Electromyogr Kinesiol,* 41**,** 124-131.

WALL-SCHEFFLER, C. M., CHUMANOV, E., STEUDEL-NUMBERS, K. & HEIDERSCHEIT, B. 2010. Electromyography activity across gait and incline: The impact of muscular activity on human morphology. *Am J Phys Anthropol,* 143**,** 601-11.

Table SI_1. Published empirically measured excitations in red; simulated activations of the human in black at 30% of the gait cycle only. Magnitudes not reported here.


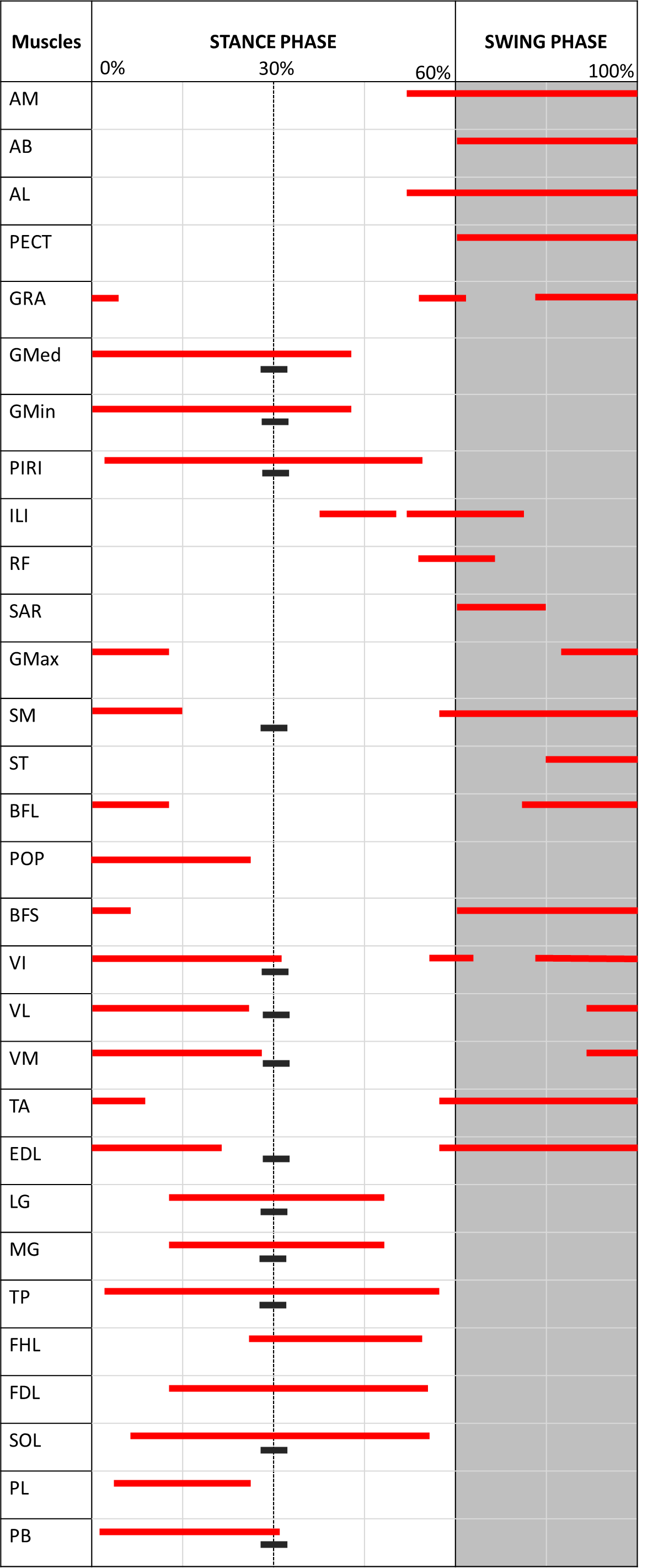

Supplement: Supplemental Information 5 — Muscle activations in the human musculoskeletal model in this study are reported at 30% of the gait cycle in comparison to published electromyography (EMG) studies. Here, simulated muscle activations as per the static optimisation outputs are used. No muscle activation dynamics have been modelled and, in this approach, simulated excitations are not produced. [file peerj-12-16821-s005.docx]
