# Supplementary figures and images for: Static versus dynamic muscle modelling in extinct species: a biomechanical case study of the Australopithecus afarensis pelvis and lower extremity
Source: PeerJ. 2024 Jan 31;12:e16821. doi: 10.7717/peerj.16821 (PMC10838096; doi:10.7717/peerj.16821)

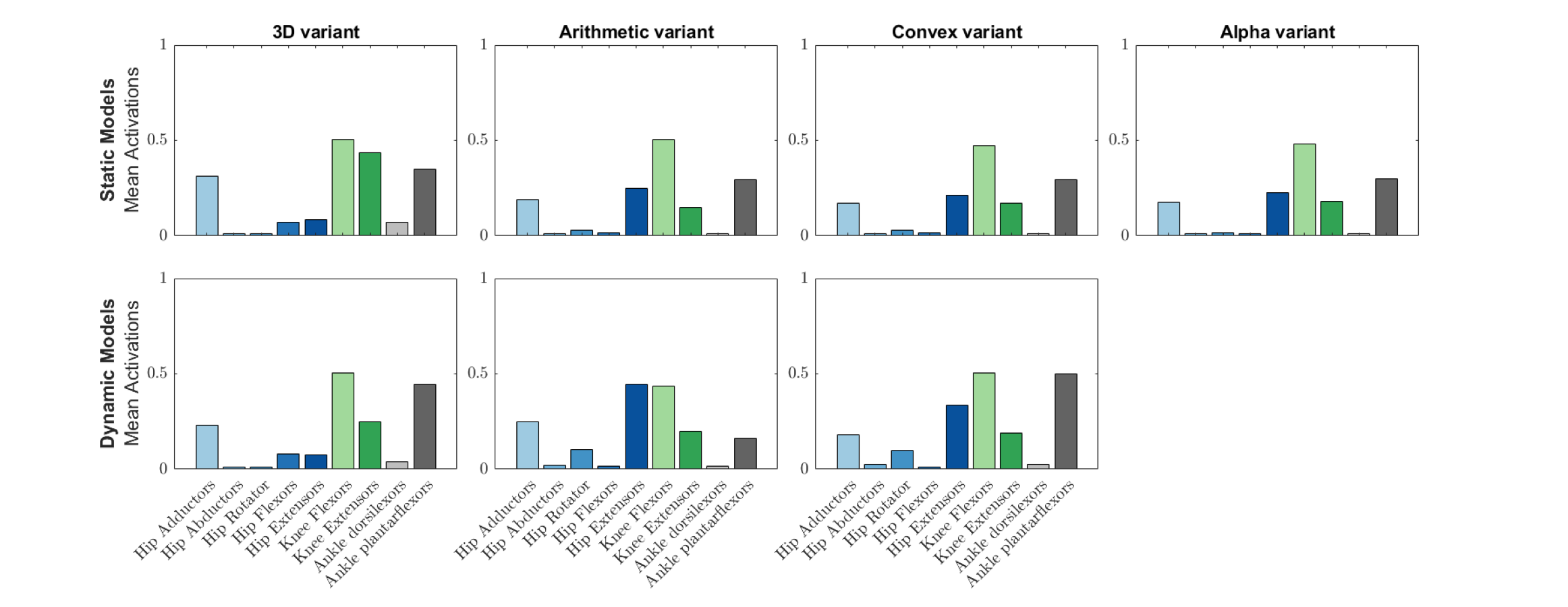

Supplement: Supplemental Information 6 — Simulated grouped mean muscle activations for the australopith models (seven variants). [file peerj-12-16821-s006.png]
